# Effects of the Interculturality and Mindfulness Program (PIM) on University Students: A Quasi-Experimental Study

**DOI:** 10.3390/ejihpe12100104

**Published:** 2022-10-05

**Authors:** Roberto Chiodelli, Saúl Neves de Jesus, Luana Thereza Nesi de Mello, Ilana Andretta, Diana Fernandes Oliveira, Maria Emília Santos Costa, Tamara Russell

**Affiliations:** 1 Research Centre for Tourism, Sustainability and Well-Being (CinTurs), 8005-139 Faro, Portugal; 2 Faculty of Human and Social Sciences (FCHS), University of Algarve (UAlg), 8005-139 Faro, Portugal; 3 Psychology Post-Graduation Program, UNISINOS University, São Leopoldo 93022-970, RS, Brazil; 4 Department of Psychology, Pontifícia Universidade Católica do Paraná (PUCPR), Curitiba 80215-901, PR, Brazil; 5 Neuroimaging Division, Institute of Psychiatry, London SE5 9NU, UK

**Keywords:** mindfulness intervention, university students, in-person group, online group, interculturality

## Abstract

Rates of mental health issues have been increasing among university students. This study investigates the effects of the Interculturality and Mindfulness Program (PIM) on academic students on mindfulness, emotional regulation, depression, anxiety, stress, life satisfaction, optimism, positive solitude, and loneliness. A quasi-experimental research was conducted, with pre- and post-test comparative measurements in three groups: in-person (IG), synchronous online (OG), and passive control (CG). A diverse group of students (n = 150; mean age = 25.4 ± 8.31) participated from two universities in Portugal. When compared to the CG, both active groups (IG and OG) demonstrated a beneficial interaction effect in acceptance, positive solitude, optimism, and mindfulness. The IG demonstrated a positive interaction effect in awareness and satisfaction with life, whereas the OG indicated a favorable interaction effect in impulse. When analyzing the intra-group effects, both active groups presented a significant improvement in stress, emotion regulation, mindfulness, positive solitude, and optimism. The OG demonstrated an improvement in awareness and loneliness. The main limitations of this research are that students were not randomly assigned, and groups were heterogeneous in nationality, education level, and sex. Nonetheless, PIM has indicated beneficial results in both IG and OG, and is a promising intervention for the prevention of mental health issues (e.g., stress, difficulties in emotional regulation, and loneliness), as well as for the promotion of well-being (e.g., positive solitude, mindfulness, life satisfaction, and optimism).

## 1. Introduction

University students’ psychological stress is increasing in terms of severity and prevalence and has become a public health concern due to the negative effects on personal development and academic performance [1]. The World Mental Health International College Student project [2], coordinated by the World Health Organization (WHO), surveyed 13,984 students from 19 universities in eight countries (spanning four continents) investigating mental disorders among first-year college students. Around one third of the participants presented at least one Diagnostic and Statistical Manual of Mental Disorders (DSM–IV) anxiety, mood, or substance disorder [2].

Mental issues, if not receiving the necessary attention, in addition to affecting student’s performance, can lead to academic dropout. Respondek et al. [3] analyzed predictors of academic success and dropout intention through a cross-sectional survey administered to 883 undergraduate students across all disciplines of a German university. The prediction of dropout intention by perceived academic control was fully mediated via anxiety, demonstrating the importance of students learning strategies to deal with this experience. Lipson and Eisenberg [4] investigated the relationship between mental health and academic performance by examining data from 3556 students at four campuses in the United States. Through multivariable models, it was found that mental health problems were a significant predictor of academic dissatisfaction and dropout intentions, while positive mental health was a significant predictor of satisfaction and persistence. In Portugal, the dropout rate of undergraduate students has increased and is currently 29% [5].

One of the factors that tends to increase anxiety in university students is the adaptation to the new context. Undergraduates encounter a social challenge as they enter a new environment and interact with a greater diversity of cultures. The University of Algarve (UAlg, Portugal), for instance, receives students from 86 different nationalities, with 25% of the student population comprising foreign students [6,7]. In addition to international scholars, there is a consistent number of students from the same country coming from different regions. Approximately 28% of Portuguese university students enter a higher education institution located outside the region of their household residence [5]. Still, other cultural varieties are found in an academic population (e.g., ethnicity, language, religion, behaviours). Therefore, students invariably undergo a process of acculturation, which occurs when an individual seeks adjustment in a new cultural context [8]. Poorly managed acculturative stress can cause feelings of marginality and alienation [9].

In order to promote social integration, interventions based on cross-cultural psychology may be valuable. Cross-cultural psychology is “the study of similarities and differences in individual psychological functioning in various cultural and ethnocultural groups” [10]. By acquiring a better knowledge of other cultures, as well as experiencing different paradigms, students may improve their intercultural competence, which is the capacity to interact adequately in cross-cultural situations, as well as in a variety of cultural contexts [11]. A wide array of interventions has been implemented to foster intercultural competence, but robust studies to assess their effects are limited [12].

In contrast, many studies with mindfulness-based interventions (MBIs) have been published as an alternative to promoting mental health, such as well-being and emotion regulation, among university students, and have demonstrated promising outcomes [13,14]. Mindfulness can be defined as a moment-to-moment, non-judgmental awareness, cultivated by paying attention deliberately [15]. MBIs have been applied since the late 70’s, when Jon Kabat-Zinn designed the Mindfulness-based Stress Reduction program (MBSR), an 8-week course. Throughout the years, the positive results of this secular, evidence-based intervention has encouraged the development of other mindfulness-based programs with different goals for either clinical or non-clinical populations [16,17,18,19].

As the implementation of group interventions seems to be a suitable initiative to promote mental health in the university setting, the Interculturality and Mindfulness Program (PIM) was developed in 2018 [20]. This program was designed to improve both interpersonal (relational) and intrapersonal (emotional) skills in academic students. Thus, the aim of this study is to examine the effects of PIM on university students in three distinct groups (in-person, online, and passive control) on the following variables: mindfulness, emotional regulation, depression, anxiety, stress, life satisfaction, optimism, positive solitude, and loneliness.

## 2. Method and Materials

The present study used a quasi-experimental design, with pre- and post-test comparative measurements in three groups: in-person (IG), online (OG), and control (CG) [21].

### 2.1. Participants

University students regularly enrolled in different courses at UAlg, located in the south of Portugal, participated in the in-person and online programs, whereas students from the University of Beja joined the control group. In total, the three groups consisted of 150 participants, with a mean age of 25.4 (SD = 8.31, minimum of 17 years old and maximum of 64 years old). Considering the participants who completed the pre- and post-test, 70 (74.3% female) belonged to the IG, 44 (90% female) were from the OG, and 36 (69.4% female) students were from the CG. Participants’ courses totalled 39, and the most frequent were: Management (24; 16%), Psychology (14; 9.3%), Water and Coast Management (10; 6.6%), Biomedical Sciences (8; 5.3%), and Computer Engineering (6; 4%). Table 1 presents data on gender, age, and nationality. As inclusion criteria, participants should attend at least four intervention sessions (66.66% attendance), being regularly enrolled in the university, and have responded to the pre-test (T1) and post-test (T2) instruments.

Participants’ socio-demographic data demonstrate homogeneity in age in the three groups, but shows heterogeneity in gender, education level, and nationality. The CG had significantly fewer women (69.4%) than the IG (74.3%) and the OG (90.9%); whereas the IG had fewer Portuguese students (25.7%) compared to the OG (75%) and the CG (83.3%). Even though most participants were undergraduates in the three groups, IG had significatively more doctorates than the OG and the CG, whereas the OG showed a higher percentage of master students than the IG and the CG.

### 2.2. Measures

*Sociodemographic questionnaire.* Developed by the authors. This is a brief form containing questions regarding participant’s sociodemographic data, as well as health and psychological conditions to certify if the program is suitable for him/her.

*Difficulties in Emotion Regulation Scale (DERS).* Developed by Gratz and Roemer [22] and translated and adapted to European Portuguese by Coutinho et al. [23]. It assesses emotional deregulation in six domains, quoted with their respective Cronbach alphas: “Non-acceptance”—nonacceptance of emotional responses (0.86), “Goals”—difficulties engaging in a goal-directed behaviour (0.85), “Impulse”—impulse control difficulties (0.80), “Awareness”—lack of emotional awareness (0.74), “Strategies”—limited access to emotion regulation strategies (0.88), and “Clarity”—lack of emotional clarity (0.75). It contains 36 items on a five-point Likert scale ranging from 1 (almost never) to 5 (almost always). The scale indicated high values of internal consistency (0.93) [23]. In the present study, total DERS Cronbach’s alpha ranged from 00.93 to 0.95. Reliability values for the subscales in this investigation were: Non-Acceptance (0.89 to 0.92), Goals (0.84 to 0.90), Impulse (0.82 to 0.90), Awareness (0.82 to 0.86), Strategies (0.87 to 0.91), and Clarity (0.78 to 0.86).

*Depression, Anxiety and Stress Scale (DASS-21).* Developed by Lovibond and Lovibond [24] and translated and adapted to European Portuguese by Apóstolo et al. [25]. DASS-21 contains a set of three Likert-type subscales, with four points ranging from 0 (“did not apply to me at all”) to 3 (“applied to me very much or most of the time”). Each subscale consists of seven items that assess the emotional states of depression, anxiety, and stress, with a maximum score of 42. Cronbach’s alpha was 0.92 for depression, 0.90 for stress, and 0.86 for anxiety. The analysis and distribution of factors among the subscales indicated that the structure of the three distinct factors was adequate. In the present study, Cronbach’s alpha values ranged from 0.82 to 0.90 for depression; from 0.77 to 0.92 for anxiety; and from 0.77 to 0.92 for stress.

*Loneliness and Positive Solitude Scale (LPSS).* Developed by Chiodelli et al. [26], this scale is a bi-dimensional, 10-item, self-report measure created to assess how often spending time with oneself generates negative or positive thoughts and sensations. The higher the scores in the loneliness dimension, the higher one’s aversion to being alone; moreover, the greater the score in the positive solitude dimension, the greater one’s perspective of being alone as something important and necessary. Cronbach’s alpha was 0.79 for loneliness and 0.85 for positive solitude. In the current study, Cronbach’s alpha values ranged from 0.75 to 0.90 for loneliness and from 0.83 to 0.88 for positive solitude.

*Philadelphia Mindfulness Scale (PHLMS).* Developed by Cardaciotto et al. [27], validated and adapted to the European Portuguese by Teixeira et al. [28]. It consists of a 5-point Likert scale and 20 items, divided in two dimensions: “Acceptance” and “Awareness.” Both dimensions presented internal consistency of 0.85 and 0.77, respectively. In the present study, Cronbach’s alpha ranged from 0.76 to 0.86 for acceptance and from 0.76 to 0.90 for awareness.

*Freiburg Mindfulness Inventory (FMI)*. Short Version by Walach et al. [29] was translated and adapted by Hirayama et al. [30]. This instrument measures mindfulness as a general construct with several interrelated facets, namely, a cognitive component, a procedural component, an experience acceptance component, and a non-acceptance component. This instrument consists of 14 items and the response format is Likert-type, with responses between 1 (“rarely”) and 4 (“almost always”). In this study, Cronbach’s alpha values ranged from 0.80 to 0.89.

*Satisfaction with Life Scale (SWLS).* Developed by Diener et al. [31], to assess the subjective judgment that each individual makes about the quality of their own life. It is a one-dimensional 5-item instrument, and the answer format is Likert-type, with answers between 1 (“strongly disagree”) and 5 (“strongly agree”), thus obtaining a minimum score of 5 (lowest satisfaction) and a maximum of 35 (highest satisfaction). It was adapted and validated in Portugal by Neto et al. [32], and the authors found an internal consistency of 0.78. In this study, Cronbach’s alpha values ranged from 0.77 to 0.87.

*Optimism Scale*. Developed by Oliveira [33] for the Portuguese population. This scale includes four items that constitute a dimension. The answer is given on an ordinal scale of 5 positions, and the answer format is Likert-type, with answers between 1 (“totally disagree”) and 5 (“totally agree”). Its internal consistency is 0.80. In this study, Cronbach’s alpha values ranged from 0.81 to 0.88.

### 2.3. Procedures

Students were invited to participate through the University’s institutional e-mail, posters, and social media. Two weeks in advance of the first session, facilitators offered a PIM presentation workshop (session zero). In session 1, participants were required to complete and sign the free and informed consent form.

In the IG, sessions were held in a spacious classroom at the University, with chairs arranged in a circle. Screen projections and a whiteboard were used as a support for the proposed activities. The OG was synchronous, conducted through Zoom video conference platform, and had the same duration as the IG. Activities which involved more physical interactions had to be adapted when transposing from IG to OG. However, the activities’ main intentions were not modified. The CG was a wait-list group for the Soft Skills for Life Program [34] from the University of Beja, also located in the south of Portugal. Therefore, the CG students answered the instruments six weeks prior to the Soft Skills Program to enroll in it and, consequently, completed the post-test before the beginning of the intervention, constituting a passive control group.

**Figure 1:**
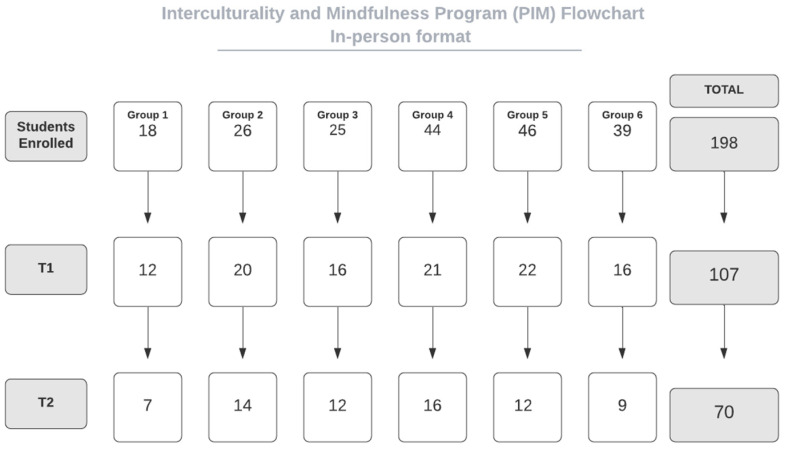
Students’ adherence in the in-person groups (IG).

**Figure 2:**
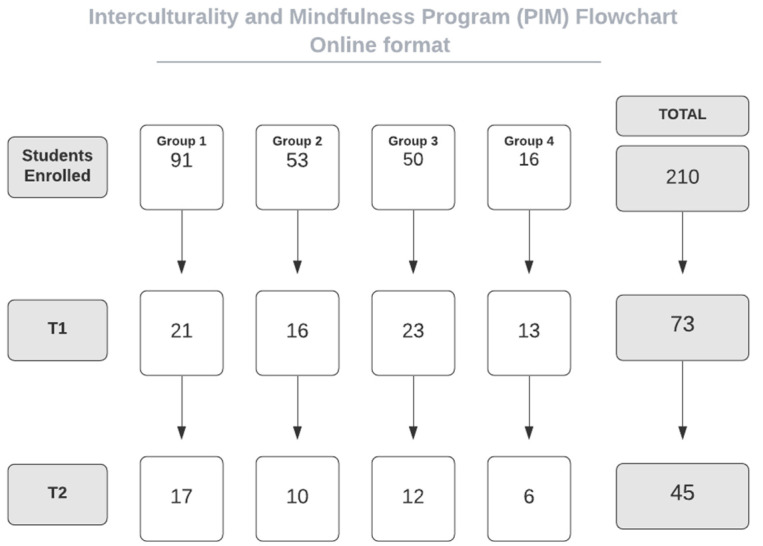
Students’ adherence in the online groups (OG).

Six PIMs’ editions were applied in the face-to face format (IG) between April 2018 to December 2019, whereas four were held via videoconference (OG) and occurred between April and December 2020. The CG was conducted between February and March 2021. Students’ adherence from both intervention formats are shown in Figure 1 and Figure 2.

#### Intervention

The Interculturality and Mindfulness program (PIM) was developed and facilitated by_Roberto Chiodelli, Diana Fernandes de Oliveira, and Luana Thereza Nesi de Mello, all Psychology doctoral students under the supervision of Saúl Neves de Jesus. Roberto Chiodelli is a psychologist, with a M.S. in Clinical Psychology, and a facilitator of the Mindfulness Protocol Body in Mind Training [35]. Diana Fernandes de Oliveira is a psychologist, with a M.S. in Health and Clinical Psychology, with advanced and specialized training in third-generation therapies. Luana Thereza Nesi de Mello is a psychologist and the head facilitator of the “Soft Skills for Life” program, offered to graduate and postgraduate courses. Most interventions were conducted in pairs, except for one, which had a single facilitator.

PIM consisted of six weekly sessions lasting two hours each (see Table 2). At the end of each meeting, through e-mail and WhatsApp (social media), participants received a summary of what was worked on, as well as the audio file for the guided meditation practices. Between each session, participants received a message of encouragement to do the weekly task practices (mindfulness), which was sent along with a short video, poem, or phrase associated to themes from the previous session.

Activities and content from the perspective of interculturality were based on an array of works in this field [36,37,38]. Most group dynamics were adapted from intercultural competence interventions, as well as playful games of psychodrama. Other activities were developed by the authors. Regarding mindfulness, the program was adapted from the book “Mindfulness: How to Find Peace in a Frantic World” [39]. Corporal practices are usually executed before formal practices of mindfulness, which occur in silence and in a static way. These body activation exercises are based on the movement exercises of the Body in Mind training protocol [35], as well as on the grounding exercise (bow and arch), a central practice of Bioenergetic Psychotherapy [40]. Further details on PIM can be found on the protocol report [20].

### 2.4. Data Collection

Psychometric tests were applied in the first and in the last (sixth) session. Whereas these instruments were answered via paper and pen in the IG, the psychometric tests, informed consent form, as well as the data protection term were completed via Google Forms in the OG. Students from the CG also filled the survey via Google Forms.

### 2.5. Data Analisys

The statistical data treatment was performed with the aid of the software Statistical Package for Social Sciences version 25.0 for Windows, with a 5% significance level. Results were presented using descriptive statistics through absolute and relative distributions (n/%), as well as through the mean and standard deviation, with the study of symmetry using the Kolmogorov–Smirnov test.

Effect analysis on the groups was performed using generalized estimating equation models, a linear model, followed by Bonferroni’s multiple comparisons test. Impact identification in each group was investigated by estimating Cohen’s d effect size.

For analysis involving the intra-group variables comparison, the generalized estimating equations (GEE) with post hoc Bonferroni was utilized. In the comparison of continuous variables between groups, the one-way variance analysis with post hoc Sheffé was used. The Student’s *t*-test was applied when comparing two independent groups.

Regarding the categorical variable comparisons between groups, Pearson’s chi-square test was used, as well as Fisher’s exact test (Monte Carlo simulation). The Cochran Q test was utilized when this analysis occurred intra-group (dependent data).

## 3. Results

**Figure 3:**
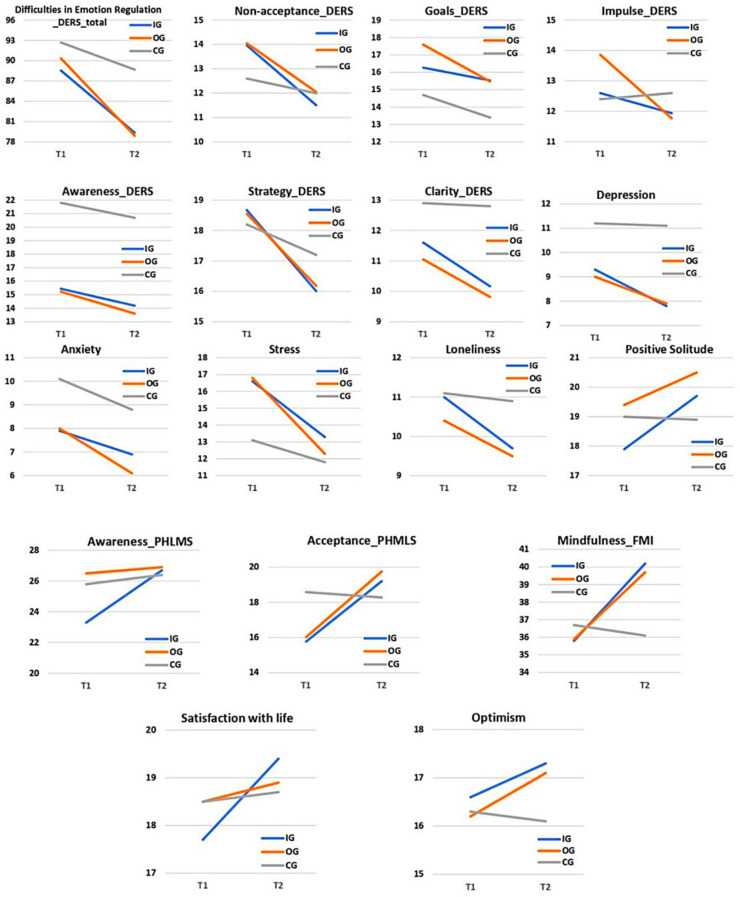
PIM’s pre- and post-test progression of each variable mean.

Generalized estimating equation (GEE) models’ data of each variable over the three groups pre- and post-intervention are found in Table 3. Three significant differences in baseline means between groups were detected. The awareness (PHMLS) baseline mean for the IG was significantly lower than the estimates of OG and CG (*p* = 0.017). In addition, goals (DERS) dimension was significantly lower in T1 for the CG when compared to IG and OG (*p* = 0.016). Lastly, the awareness (DERS) baseline mean was higher in CG when associated with the two other groups (*p* ≤ 0.001). Figure 3 presents the evolution of each dimension in the three groups.

Table 4 presents variables that demonstrated a significant difference in interaction effect, indicating that the groups’ mean scores, along the pre- and post-test, had distinct behaviors. An interaction effect was detected in the awareness dimension (chi-square Wald = 12.996; *p* = 0.002), where IG (*p* = 0.002; d = 0.548) showed a significant growth over time, whereas OG (*p* = 0.168) and CG (*p* = 0.502) means remained unchanged. All interaction effects were in a positive direction.

Table 5 demonstrates the variables that showed significant intra-group differences between PIM’s pre- and post-test. Once more, all differences found were beneficial to the participant. The IG exclusively demonstrated improvements in two variables—Awareness (PHMLS) and Satisfaction with life (SWLS)—whereas the OG presented exclusive outcomes on three: Impulse (DERS), Awareness (DERS), and Loneliness (LPSS).

## 4. Discussion

These findings demonstrate that, compared to the passive control group, both in-person (IG) and online (OG) PIM had a positive impact on most variables analyzed. In general, IG and OG were equivalent in terms of their effects. IG presented two specific dimensions of interaction effect (Awareness (PHMLS) and Satisfaction with Life (SWLS)) versus one for the OG (Impulse (DERS)). On the other hand, OG had three specific intra-group beneficial effects (Impulse (DERS), Awareness (DERS), and Loneliness (LPSS)), versus two of the IG (Awareness (PHMLS) and Satisfaction with Life (SWLS)). A more detailed comparative analysis between the IG and OG was performed in the study which examines PIM effects in three different times (pre-test, post-test, and follow-up test) [41].

Anxiety (DASS-21) and depression (DASS-21) are the variables that did not demonstrate significant effects in any group. Although some research with MBIs among university students demonstrates significant reductions in depression and anxiety [42,43], other studies have found non-significant reductions in these dimensions [44]. The fact that the current research sample is a non-clinical population may reduce the intervention’s impact on depression and anxiety, as significant reductions tend to occur in populations that already confer high levels of depression and anxiety. Controlled studies indicate MBCT may be effective in reducing depressive symptoms among individuals with acute depression, and meta-analyses indicate MBIs significantly reduce anxiety among populations with anxiety disorder [45]. Considering this program has a shorter duration (6 weeks) compared to traditional MBIs (8 weeks), an analysis of PIM follow-up outcomes (within 3 months after the end of intervention) is suggested [41].

As found in other investigations [46,47], there was an increase in mindfulness skills after the interventions. Both in the IG and in the OG, there was a higher mindfulness dimension score (FMI), which means present moment observation without judging and openness to negative experience [29], as well as a higher Acceptance variable (PHMLS), which is an attitude of openness, free from defenses, beliefs, and interpretations of one’s own internal or external experience [27]. However, only the IG showed an increase in the Awareness dimension (PHMLS), which refers to monitoring the internal or external experience as it occurs. A hypothesis for such a result may be that the IG enables more bodily dynamics than the OG, since the body is one of the most accessible ways to develop awareness [19].

Mindfulness skills have a direct relationship with emotion regulation [48], which is a very broad concept. Gratz and Roemer [22] argue that emotion regulation involves (a) acceptance, awareness, and understanding of emotions, (b) the ability to control impulsive behaviors and to behave in accordance with desired goals when negative emotions are experienced, and (c) the ability to use appropriate emotional adjustment strategies to meet individual goals and situational demands. This research outcome reveals a significant decrease in emotion regulation difficulties in both intervention groups, confirming other reported findings in the literature. Shahidi et al. [49] assessed fifty students randomly divided into experimental (MBSR) and control groups. Their results showed that the MBSR program has had steady favorable effects on emotion regulation.

When analyzing the intra-group results of both PIM formats, all DERS subscales presented a significant reduction in at least one of the groups. Impulse had a significant interaction effect for the OG, which denotes that the online intervention more sharply reduced the difficulty of remaining in control of participant’s behavior when experiencing negative emotions, when compared to the other groups. The OG also showed an intra-group reduction in awareness (DERS), which has a more specific meaning than the PHMLS variable of the same name. It refers to a lack of awareness or attention to emotional responses, whereas the PHMLS dimension involves external awareness, thoughts, bodily sensation, as well as emotions. The variables Strategy (belief that there is little one can do to regulate oneself once upset), Clarity (the extent to which an individual is unclear about his or her emotions), and Non-acceptance (tendency to have a negative secondary or non-accepting reaction to one’s own distress) decreased in both OG and IG. Lastly, the goals dimension, which means the difficulty in concentrating and/or accomplishing tasks when experiencing negative emotions, showed a reduction in the OG and, interestingly, in the CG, being the only variable with a significant change in the passive group.

An interaction effect for Positive Solitude was found in both active groups. This construct can be defined as a voluntary aloneness, during which personality development and creativity may emerge. In this state, the individual enjoys the experience of spending solitary time and can use it to explore himself/herself. He/she is not avoiding social interaction due to social anxiety or preference [50,51]. Basically, through solitary practices with an intention to observe one’s emotions and thoughts with acceptance and self-compassion, mindfulness may have influenced students to feel more comfortable in being by themselves [52]. The study also identified a significant reduction in the dimension of loneliness (aversion to being alone) in the OG. Loneliness is a public health issue and has become more critical with the need for social isolation imposed by COVID-19 [53]—specifically, the period when OGs were implemented.

Stress reduction in both active groups is relevant, but expected when examining the MBI literature. Lovibond and Lovibond [24] define stress as relaxation difficulty, nervous excitement, impatience, irritation, and reactivity. Consistent with this finding, a growing number of RCTs show that MBIs positively impact stress-related aspects of physical health, ranging from chronic pain, immune system functioning, specific diseases symptoms, and healthy behaviors [54].

Optimism also showed a significant interaction effect in the two active groups when compared to the passive one. This dimension can be defined as an emotional and cognitive predisposition to think and react to others and events in a favorable way, instead of expecting harmful outcomes [55]. Numerous studies demonstrate that positive ideas about the future predicts coping. Vizoso et al. [56] examined the relationship between coping strategies, dispositional optimism, academic burnout, and academic performance of 532 Spanish undergraduate students. Emotional exhaustion was significantly and negatively predicted by optimism.

Satisfaction with life, another positive dimension, had a significant interaction effect in the IG. This variable refers to the cognitive component of subjective well-being, in which individuals globally assess the quality of their lives based on their own criteria [31]. By the end of the programs, as well as in the follow-up test meetings, students expressed that the skills learned in PIM improved the way they dealt with difficulties. Existing research reveals that self-esteem, depression, positive and negative effects, family structure, happiness, physical, psychological, and social health are considered predictors of general life satisfaction [57].

In addition to mindfulness, intercultural-based dynamics had an important impact on the participants’ journey along PIM. These activities fostered a sharing of experiences among students based on the topic of cultural diversity, to provide greater group cohesion, which is a key component for all groups [58]. Participants used to comment on the importance of listening to others and being able to express themselves, which allowed a greater integration in the university context. Recommendations for future studies with the PIM include the analysis of group cohesion and intercultural competence.

When analyzing student’s adherence (Figure 1 and Figure 2), it is noticed that drop-out rates were substantially greater in the OG than in the IG between enrollment and pre-test (T0–T1). This may have occurred since registrations in the online groups were disseminated more widely over the internet than in the in-person groups. On the other hand, in the post- and follow-up tests ratio (T2–T2), which is presented in a study that compared both interventions [41], the IG had a higher drop-out rate than the OG. The higher difficulty for participants to be present at the follow-up in-person meetings might have been the main reason for this.

Some limitations are detected in this study and should be considered. Although groups were homogeneous in age, they were heterogeneous in terms of gender, education level, and nationality. The nationality difference was since the first IGs were more publicized to international students. Moreover, group application occurred at different times. Furthermore, COVID-19 affected everyone and should also be taken into consideration. Another limitation is that participants were not randomly allocated to the three groups, which would offer greater reliability to the results. Finally, the applied measures were self-reported. The use of biological or behavioral measures would offer more robust evidence.

## 5. Conclusions

In spite of its limitations, this study provides evidence that confirms results of similar interventions and reinforces the importance of programs to be implemented in the academic environment. PIM has proved to be relevant and very promising both for the prevention of mental health problems (stress, difficulties in emotional regulation, loneliness) and for the promotion of well-being (positive solitude, mindfulness, life satisfaction, optimism). Such benefits tend to promote a higher engagement of the student in the university context and, consequently, reduce academic dropout. We hope PIM can be replicated in future studies.

## Figures and Tables

**Table 1:** General sample characterization.

Variables		Groups			
	Total (*n* = 150)	In-Person (IG) (*n* = 70)	Online (OG) (*n* = 44)	Control (CG) (*n* = 36)	*p*
	*n* (%)				
Sex					0.041 ^2^
Male	33 (22)	18 (25.7)	4 (9.1)	11 (30.6)	
Female	117 (78)	52 (74.3)	40 (90.9)	25 (69.4)	
Age ^1^	25.5 ± 8.3	26.3 ± 7.7	24.1 ± 6.2	25.6 ± 11.2	0.269 ^3^
Nationality					<0.001 ^4^
Brazilian	65 (43.3)	52 (74.3)	11 (25.0)	2 (5.6)	
Portuguese	81 (54)	18 (25.7)	33 (75.0)	30 (83.3)	
Cape Verdean and Mozambican	4 (2.7)	-	-	4 (11.2)	
Education					0.033
Higher Professional Technical Courses	3 (2)	1 (1.4)	-	2 (5.5)	
Undergraduate	108 (72)	47 (67.1)	30 (68.1)	31 (86.1)	
Master	27 (18)	13 (18.5)	11 (25)	3 (8.3)	
Doctorate	12 (8)	9 (12.8)	3 (6.8)	-	

*Note.* ^1^ Missing Data (*n* = 8; 5.3%); ^2^ Pearson’s chi-square test; ^3^ One-way ANOVA; ^4^ Fisher’s exact test.

**Table 2:** Interculturality and Mindfulness Program (PIM) overview.

	Activities
(0) Program Presentation	Welcoming; “ice-breaker”: ball game; program presentation; mindfulness practice
(1) Introduction and group integration “Land in sight: welcome to the University environment!”/Being present	Facilitators and program introduction; “ice-breakers”: planning seats and rotatory interviews; “culture shock” activity; mindfulness presentation and body scan
(2) Positive Intercultural Attitude I “Anchorage”/Mindfulness in the daily routine	“Sharing”; “warm-up”: “Rá” game; “ice-breaker”: three sentences activity; difficulties and strategies to acculturation; informal mindfulness meditation: mindful eating practice
(3) Positive Intercultural Attitude II “(Re) Socialization”/Body and Emotions	“Sharing”; “warm-up”: imaginary objects activity; cultural knowledge: chocolate game; stages of cultural adaptation; acceptance; emotions in the body practice
(4) Intercultural Communication I “Verbal and nonverbal communication”/Self Compassion	“Sharing”; “warm-up”: 1, 2, 3 game; behavioral differences in communication; self-compassion; walking mindfully practice; loving-kindness meditation
(5) Intercultural Communication II “What do we have in common?”/Observing thoughts and Gratitude	“Sharing”; warm-up: “Pim game” and “Hot Potato”; “proverb’s game”; group bubbles; observing thoughts; gratitude
(6) Program Completion “Weaving the Support Network”/Week 6 is the rest of our lives	“Sharing”; warm-up: “weaving connections activity”; social support network; rhythmic breath meditation; “week 6 is the rest of our lives”; final celebration

**Table 3:** Generalized estimating equation (GEE) models for evaluating the PIM effect over time.

Evaluations in Time	Groups							Interaction Effects (GEE) ^C^
	In-Person (IG) (*n* = 70)		Online (OG) (*n* = 44)		Control (CG) (*n* = 36)		Between Groups B	
	M	SD	M	SD	M	SD		
T1_Awareness (PHMLS)	23.3 ^b^	6.1	26.5 ^a^	5.8	25.8 ^a^	6.3	0.017	0.002
T2_Awareness (PHMLS)	26.7	6.3	26.9	4.1	26.4	5.8	0.906	
Intra-group A	0.002		0.168		0.502			
T2–T1	Δ = 3.4; d = 0.548		Δ = 0.4; d = 0.081		Δ = 0.6; d = 0.099			
T1_Acceptance (PHMLS)	15.8	7.5	16.0	6.6	18.6	6.9	0.113	<0.001
T2_Acceptance (PHMLS)	19.2	6.4	19.8	5.3	18.3	6.7	0.537	
Intra-group ^A^	<0.001		0.002		0.859			
T2–T1	Δ = 3.4; d = 0.489		Δ = 3.7; d = 0.622		Δ = −0.3; d = −0.044			
T1_Non-accept (DERS)	14.0	6.1	14.1	5.6	12.6	6.2	0.491	0.158
T2_Non-accept (DERS)	11.5	6.3	12.1	5.7	12.0	5.4	0.849	
Intra-group ^A^	0.001		0.029		0.456			
T2–T1	Δ = −2.5; d = −0.403		Δ = −2.0; d = −0.354		Δ = −0.6; d = −0.103			
T1_Goals (DERS)	16.3 ^a^	4.5	17.6 ^a^	4.8	14.7 ^b^	3.8	0.016	0.190
T2_Goals (DERS)	15.5	4.5 ^a^	15.5 ^a^	4.1	13.4 ^b^	3.8	0.045	
Intra-group ^A^	0.143		0.001		0.039			
T2–T1	Δ = −0.7; d = −0.156		Δ = −2.1; d = −0.472		Δ = −1.3; d = −0.342			
T1_ Impulse (DERS)	12.6	5.0	13.9	4.8	12.4	3.9	0.295	0.025
T2_ Impulse (DERS)	11.9	4.9	11.8	3.8	12.6	4.7	0.655	
Intra-group ^A^	0.209		0.002		0.710			
T2–T1	Δ = −0.7; d = −0.141		Δ = −2.1; d = −0.488		Δ = 0.2; d = 0.051			
T1_Awareness (DERS)	15.4 ^b^	5.1	15.2 ^b^	4.7	21.8 ^a^	5.1	<0.001	0.892
T2_ Awareness (DERS)	14.2 ^b^	5.1	13.6 ^b^	4.7	20.7 ^a^	4.8	<0.001	
Intra-group ^A^	0.051		0.040		0.068			
T2–T1	Δ = −1.3; d = −0.255		Δ = −1.6; d = −0.340		Δ = −1.1; d = −0.222			
T1_Strategy (DERS)	18.7	7.1	18.6	6.3	18.2	6.5	0.939	0.254
T2_Strategy (DERS)	16.0	7.1	16.2	6.4	17.2	4.8	0.602	
Intra-group ^A^	<0.001		0.026		0.263			
T2–T1	Δ = −2.7; d = −0.380		Δ = −2.4; d = −0.338		Δ = −1.0; d = −0.177			
T1_Clarity (DERS)	11.6	4.0	11.1	3.1	12.9	1.7	0.057	0.064
T2_Clarity (DERS)	10.2 ^b^	4.0	9.8 ^b^	3.3	12.8 ^a^	1.5	<0.001	
Intra-group ^A^	0.001		0.042		0.679			
T2–T1	Δ = −1.4; d = −0.350		Δ = −1.2; d = −0.375		Δ = −0.1; d = −0.063			
T1_DERS total	88.5	22.8	90.3	21.3	92.7	18.4	0.658	0.131
T2_DERS total	79.4 ^b^	23.6	78.9 ^b^	19.7	88.7 ^a^	16.6	0.044	
Intra-group ^A^	<0.001		0.002		0.118			
T2–T1	Δ = −9.2; d = −0.397		Δ = −11.4; d = −0.566		Δ = −4.0; d = −0.229			
T1_Loneliness	11.0	3.8	10.4	3.5	11.1	4.2	0.705	0.393
T2_Loneliness	9.7	3.2	9.5	3.4	10.9	3.9	0.215	
Intra-group ^A^	0.060		0.041		0.724			
T2–T1	Δ = −1.3; d = −0.371		Δ = −0.9; d = −0.261		Δ = −0.2; d = −0.049			
T1_P. Solitude	17.9	4.6	19.4	3.6	19.0	3.9	0.353	0.013
T2_P. Solitude	19.7	3.7	20.5	3.5	18.9	3.3	0.126	
Intra-group ^A^	0.039		0.001		0.873			
T2–T1	Δ = 1.8; d = 0.434		Δ = 1.1; d = 0.310		Δ = −0.1; d = −0.028			
T1_SWLS	17.7	4.0	18.5	4.1	18.5	4.1	0.441	0.017
T2_SWLS	19.4	3.9	18.9	3,8	18.7	4.2	0.665	
Intra-group ^A^	<0.001		0.278		0.737			
T2–T1	Δ = 1.7; d = 0.430		Δ = 0.4; d = 0.101		Δ = 0.2; d = 0.048			
T1_Optimism	16.6	3.5	16.2	3.2	16.3	2.6	0.784	0.019
T2_Optimism	17.3	2.8	17.1	2.6	16.1	2.3	0.062	
Intra-group ^A^	0.010		0.012		0.502			
T2–T1	Δ = 0.78; d = 0.222		Δ = 0.98; d = 0.310		Δ = −0.2; d = −0.081			
T1_Mindfulness	35.8	8.3	35.9	7.1	36.7	7.6	0.855	<0.001
T2_Mindfulness	40.2 ^a^	6.8	39.7 ^a^	5.9	36.1 ^b^	7.1	0.008	
Intra-group ^A^	<0.001		0.005		0.469			
T2–T1	Δ = 4.4; d = 0.582		Δ = 3.8; d = 0.584		Δ = −0.6; d = −0.154			
T1_Depression	9.3	7.9	9.0	9.2	11.2	9.7	0.481	0.739
T2_Depression	7.8	7.0	7.9	6.8	11.1	10.2	0.097	
Intra-group ^A^	0.144		0.393		0.972			
T2–T1	Δ = −1.5; d = −0.201		Δ = −1.1; d = −0.137		Δ = −0.1; d = −0.483			
T1_Anxiety	7.9	7.9	8.0	8.2	10.1	9.5	0.414	0.850
T2_Anxiety	6.9	6.8	6.1	6.8	8.8	9.3	0.256	
Intra-group ^A^	0.238		0.133		0.447			
T2–T1	Δ = −1.0; d = −0.136		Δ = −1.9; d = −0.253		Δ = −1.3; d = −0.484			
T1_Stress	16.6	9.0	16.8	11.2	13.1	8.8	0.147	0.193
T2_Stress	13.3	7.4	12.3	7.4	11.8	8.8	0.791	
Intra-group ^A^	0.004		0.002		0.349			
T2–T1	Δ = −3.3; d = −0.402		Δ = −4.5; d = 0.484		Δ = −1.3; d = −0.484			

*Note*. ^A^: Intra-group mean comparison, EEG between times with post hoc Bonferroni; ^B^: Mean comparison between groups, ANOVA (one way) with post hoc Bonferroni, where means followed by equal lowercase letters (^ab^) do not differ at a significance of 5%; ^C^: EEG, linear model for effects of time, group, and interaction with post hoc Bonferroni; Δ: variation between mean scores; d: Cohen’s d effect size.

**Table 4:** Variables with significant interaction effects in each group.

		T1–T2	
		X^2^_Wald_	*p*
IG x OG and CG	Awareness (PHLMS)	12.996	0.002
	Satisfaction with life (SWLS)	7.996	0.017
OG x IG and CG	Impulse (DERS)	7.403	0.025
IG and OG x CG	Acceptance (PHLMS)	17.573	<0.001
	P. Solitude (LPSS)	8.640	0.13
	Optimism	7.935	0.019
	Mindfulness (FMI)	22.315	<0.001

*Note*. Groups in bold are the ones that showed a significant interaction effect. X^2^_Wald_: chi square Wald. *p*: significance minimum level.

**Table 5:** Variables with difference between PIM’s pre- and post-test divided by groups.

Significant Difference Intra-Groups (T1–T2)	
	Variables (*d*)
In-person group (IG)	Awareness (PHLMS) (0.54),
	Acceptance (PHLMS) (0.48),
	Non-acceptance (DERS) (0.40),
	Strategy (DERS) (0.38),
	Clarity (DERS) (0.35),
	DERS total (0.39),
	P. Solitude (0.43),
	Satisfaction with life (SWLS) (0.43),
	Optimism (0.22),
	Mindfulness (FMI) (0.58),
	Stress (DASS-21) (0.40)
Online group (OG)	Acceptance (PHLMS) (0.62),
	Impulse (DERS) (0.48),
	Non-acceptance (DERS) (0.35),
	Goals (DERS) (0.47),
	Awareness (DERS) (0.34),
	Strategy (DERS) (0.33),
	Clarity (DERS) (0.37),
	DERS total (0.56),
	P. Solitude (LPSS) (0.31),
	Loneliness (LPSS) (0.26),
	Optimism (0.31),
	Mindfulness (FMI) (0.58),
	Stress (DASS-21) (0.48)
Control group (CG)	Goals (DERS) (0.34)

*Note*. *d* = Cohen’s d effect size. Variables in bold: variables were significantly different in one exclusive group.

## Data Availability

The datasets generated during and/or analyzed during the current study are available from the corresponding author on reasonable request.

## References

[1] Hohenshil T.H., Amundson N.E., Niles S.G. (2013). Counseling around the World: An International Handbook.

[2] Auerbach R.P., Mortier P., Bruffaerts R., Alonso J., Benjet C., Cuijpers P., Demyttenaere K., Ebert D.D., Green J.G., Hasking P. (2018). WHO World Mental Health Surveys International College Student Project: Prevalence and distribution of mental disorders. J. Abnorm. Psychol..

[3] Respondek L., Seufert T., Stupnisky R., Nett U.E. (2017). Perceived Academic Control and Academic Emotions Predict Undergraduate University Student Success: Examining Effects on Dropout Intention and Achievement. Front. Psychol..

[4] Lipson S.K., Eisenberg D. (2018). Mental health and academic attitudes and expectations in university populations: Results from the healthy minds study. J. Ment. Health.

[5] Direção-Geral de Estatísticas da Educação e Ciência (DGEEC), Direção de Serviços de Estatísticas da Educação (DSEE) (2019). Estatísticas da Educação 2018/2019.

[6] Público (2019). Mais de 20% dos Alunos da Universidade do Algarve são Estrangeiros. https://www.publico.pt/2019/11/25/p3/noticia/quinto-alunos-universidade-algarve-sao-estrangeiros-1894965.

[7] Rodrigues H. (2019). Em 2020, um Quarto dos Alunos da Universidade do Algarve Serão Estrangeiros. https://www.sulinformacao.pt/2019/12/em-2020-um-quarto-dos-alunos-da-universidade-do-algarve-serao-estrangeiros/.

[8] Jacob L.M. (2020). Acculturation.

[9] Berry J.W., Kim U., Minde T., Mok D. (1987). Comparative Studies of Acculturative Stress. Int. Migr. Rev..

[10] Berry J.W., Poortinga Y.H., Segall M.H., Dasen P.R. (2002). Cross-Cultural Psychology: Research and Applications.

[11] Bennett J.M., Bennett M.J., Landis D., Bennett J.M., Bennett M.J. (2004). Developing intercultural sensitivity: An integrative approach to global and domestic diversity. Handbook of Intercultural Training.

[12] Zhang X., Zhou M. (2019). Interventions to promote learners’ intercultural competence: A meta-analysis. Int. J. Intercult. Relat..

[13] Chiodelli R., de Mello L.T.N., de Jesus S.N., Beneton E.R., Russel T., Andretta I. (2020). Mindfulness-based interventions in undergraduate students: A systematic review. J. Am. Coll. Health.

[14] Dawson A.F., Brown W.W., Anderson J., Datta B., Donald J.N., Hong K., Allan S., Mole T.B., Jones P.B., Galante J. (2020). Mindfulness-Based Interventions for University Students: A Systematic Review and Meta-Analysis of Randomised Controlled Trials. Appl. Psychol. Health Well-Being.

[15] Kabat-Zinn J. (2015). Mindfulness. Mindfulness.

[16] Chiodelli R., Mello L.T.N., Jesus S.N., Andretta I. (2018). Effects of a brief mindfulness-based intervention on emo-tional regulation and levels of mindfulness in senior students. Psicol. Reflexão Crítica.

[17] Williams J.M.G., Russell I., Russell D. (2008). Mindfulness-based cognitive therapy: Further issues in current evidence and future research. J. Consult. Clin. Psychol..

[18] Cusens B., Duggan G.B., Thorne K., Burch V. (2010). Evaluation of the breathworks mindfulness-based pain management programme: Effects on well-being and multiple measures of mindfulness. Clin. Psychol. Psychother..

[19] Russell T. (2011). Body in mind training: Mindful movement for severe and enduring mental illness. Br. J. Wellbeing.

[20] Chiodelli R., Mello L.T.N., Jesus S.N., Oliveira D.F., Russel T., Andretta I. (2021). Interculturality and Mindfulness Program (PIM): A Protocol Report of an Intervention with University Students.

[21] Creswell J.W., Creswell J.D. (2018). Research Design: Qualitative, Quantitative and Mixed Methods Approaches.

[22] Gratz K.L., Roemer L. (2004). Multidimensional Assessment of Emotion Regulation and Dysregulation: Development, Factor Structure, and Initial Validation of the Difficulties in Emotion Regulation Scale. J. Psychopathol. Behav. Assess..

[23] Coutinho J., Ribeiro E., Ferreirinha R., Dias P. (2010). Versão portuguesa da escala de dificuldades de regulação emocional e sua relação com sintomas psicopatológicos. Arch. Clin. Psychiatry.

[24] Lovibond S.H., Lovibond P.F. (1995). Manual for the Depression Anxiety Stress Scales.

[25] Apóstolo J.L.A., Mendes A.C., Azeredo Z.A. (2006). Adaptation to Portuguese of the Depression, Anxiety and Stress Scales (DASS). Rev. Lat.-Am. Enferm..

[26] Chiodelli R., Mello L.T.N., Jesus S.N., Viseu J., Russel T., Andretta I. (2021). Loneliness and Positive Solitude Scale (LPSS): How Does It Feel to Be Alone with Yourself? A Portuguese Scale Validation.

[27] Cardaciotto L., Herbert J.D., Forman E., Moitra E., Farrow V. (2008). The assessment of present-moment awareness and acceptance: The Philadelphia Mindfulness Scale. Assessment.

[28] Teixeira R.J., Ferreira G., Pereira M.G. (2017). Portuguese validation of the Cognitive and Affective Mindfulness Scale-Revised and the Philadelphia Mindfulness Scale. Mindfulness Compassion.

[29] Walach H., Buchheld N., Buttenmüller V., Kleinknecht N., Schmidt S. (2006). Measuring mindfulness—The Freiburg Mindfulness Inventory (FMI). Pers. Individ. Differ..

[30] Hirayama M.S., Milani D., Rodrigues R.C.M., De Barros N.F., Alexandre N.M.C. (2014). A percepção de comportamentos relacionados à atenção plena e a versão brasileira do Freiburg Mindfulness Inventory. Ciência Saúde Coletiva.

[31] Diener E., Emmons R.A., Larsen R.J., Griffin S. (1985). The Satisfaction with Life Scale. J. Personal. Assess..

[32] Neto F., Barros J.H., Barros A., Almeida L., Santiago R., Silva P., Caetano O., Marques J. (1990). Satisfação com a vida. Acção Educativa: Análise Psico-Social.

[33] Barros de Oliveira J.H. (1998). Optimismo: Teoria e avaliação (proposta de uma nova escala). Psicol. Educ. Cult..

[34] Mello L.T.N. (2021). Desenvolvimento e Avaliação de uma Intervenção Online de Competências Transversais com Universitários.

[35] Russell T. (2015). Mindfulness in Motion.

[36] Sam D.L., Berry J.W. (2016). Cambridge Handbook of Acculturation Psychology.

[37] Deardorff D.K., De Wit H., Heyl J.D., Adams T. (2012). The SAGE Handbook of International Higher Education.

[38] Sebben A. (2013). Cultural Exchange Program: Understand It & Fall for It.

[39] Williams M., Penman D. (2015). Atenção Plena: Como Encontrar a paz em um Mundo Frenético.

[40] Lowen A., Lowen L. (1977). Exercícios de Bioenergética: O Caminho para uma Saúde Vibrante.

[41] Chiodelli R., Mello L.T.N., Jesus S.N., Oliveira D.F., Russel T., Andretta I. (2021). Effects of a Mindfulness-Based Intervention: A Comparison between a Synchronous Online and an In-Person Program.

[42] Haukaas R.B., Gjerde I.B., Varting G., Hallan H.E., Solem S. (2018). A Randomized Controlled Trial Comparing the Attention Training Technique and Mindful Self-Compassion for Students With Symptoms of Depression and Anxiety. Front. Psychol..

[43] Panahi F., Faramarzi M. (2016). The Effects of Mindfulness-Based Cognitive Therapy on Depression and Anxiety in Women with Premenstrual Syndrome. Depress. Res. Treat..

[44] Ko C.M., Grace F., Chavez G.N., Grimley S.J., Dalrymple E.R., Olson L.E. (2018). Effect of Seminar on Compassion on student self-compassion, mindfulness and well-being: A randomized controlled trial. J. Am. Coll. Health.

[45] Strauss C., Cavanagh K., Oliver A., Pettman D. (2014). Mindfulness-Based Interventions for People Diagnosed with a Current Episode of an Anxiety or Depressive Disorder: A Meta-Analysis of Randomised Controlled Trials. PLoS ONE.

[46] Vibe M., Solhaug I., Rosenvinge J.H., Tyssen R., Hanley A., Garland E. (2018). Six-year positive effects of a mindfulness-based intervention on mindfulness, coping and well-being in medical and psychology students; Results from a randomized controlled trial. PLoS ONE.

[47] Gu Y., Xu G., Zhu Y. (2018). A Randomized Controlled Trial of Mindfulness-Based Cognitive Therapy for College Students with ADHD. J. Atten. Disord..

[48] Hill C.L.M., Updegraff J.A. (2012). Mindfulness and its relationship to emotional regulation. Emotion.

[49] Shahidi S., Akbari H., Zargar F. (2017). Effectiveness of mindfulness-based stress reduction on emotion regulation and test anxiety in female high school students. Int. J. Health Promot. Educ..

[50] Galanaki E. (2004). Are children able to distinguish among the concepts of aloneness, loneliness, and solitude?. Int. J. Behav. Dev..

[51] Storr A. (1988). Solitude: A Return to the Self.

[52] Leavitt C.E., Butzer B., Clarke R.W., Dvorakova K., Coplan R.J., Bowker J.C., Nelson L.J. (2021). Intentional Solitude and Mindfulness. The Handbook of Solitude.

[53] Killgore W.D., Cloonan S.A., Taylor E.C., Dailey N.S. (2020). Loneliness: A signature mental health concern in the era of COVID-19. Psychiatry Res..

[54] Creswell J.D. (2017). Mindfulness Interventions. Annu. Rev. Psychol..

[55] Mehrabian A. (1998). Manual for the Self Esteem and Optimism-Pessimism Scales.

[56] Vizoso C., Arias-Gundín O., Rodríguez C. (2019). Exploring coping and optimism as predictors of academic burnout and performance among university students. Educ. Psychol..

[57] Marques J.R.V. (2011). A Influência de um Curso de Meditação nos Níveis de Mindfulness, Satisfação com a Vida e Optimismo. Master’s Thesis.

[58] Marmarosh C.L., Sproul A., Parks C.D., Tasca G.A. (2021). Group cohesion: Empirical evidence from group psychotherapy for those studying other areas of group work. The Psychology of Groups: The Intersection of Social Psychology and Psychotherapy Research.

